# Osteoblastic skeletal metastases mimicking high–bone-turnover metabolic bone disease in gastric adenocarcinoma

**DOI:** 10.1210/jcemcr/luag269

**Published:** 2026-09-16

**Authors:** Sai Meghana Madala, Asha Ranjan, Venkateswarlu Damacharlla, Sadhanandham Shanmugasundaram, Venkata Sai Pulivadula Mohanarangam, Leena Dennis Joseph

**Affiliations:** Department of Endocrinology and Metabolism, Sri Ramachandra Institute of Higher Education and Research, Chennai 600116, India; Department of Endocrinology and Metabolism, Sri Ramachandra Institute of Higher Education and Research, Chennai 600116, India; Department of Endocrinology and Metabolism, Sri Ramachandra Institute of Higher Education and Research, Chennai 600116, India; Department of Cardiology, Sri Ramachandra Institute of Higher Education and Research, Chennai 600116, India; Department of Radiology, Sri Ramachandra Institute of Higher Education and Research, Chennai 600116, India; Department of Pathology, Sri Ramachandra Institute of Higher Education and Research, Chennai 600116, India

**Keywords:** high bone turnover, osteoblastic metastases, gastric adenocarcinoma

## Abstract

Markedly elevated bone-specific alkaline phosphatase with radiographic skeletal abnormalities is a common endocrine presentation, most often attributed to disorders with high bone turnover, including Paget disease of bone or osteomalacia. Rarely, tumor-driven osteoblastic metastases may closely mimic this biochemical and radiographic phenotype, thereby potentially delaying the diagnosis of occult malignancy. This report describes a 50-year-old woman presenting with progressive dyspnea and constitutional symptoms who was found to have massive pericardial effusion with cardiac tamponade requiring pericardiocentesis. Imaging revealed diffuse osteosclerotic lesions with axial skeleton involvement. Laboratory examination showed markedly elevated alkaline phosphatase with a disproportionately high bone-specific component, alongside mild hypocalcemia, secondary hyperparathyroidism, and severe vitamin D deficiency, initially suggesting a high–bone-turnover metabolic bone disorder. However, whole-body fluorine-18 fluorodeoxyglucose positron emission tomography/computed tomography (^18^F-FDG PET/CT) demonstrated multiple sclerotic skeletal lesions with mild homogeneous marrow uptake, suggesting metastatic disease. Upper gastrointestinal endoscopy identified a lesion in gastric antrum, and biopsy confirmed poorly differentiated signet-ring cell adenocarcinoma. Bone marrow biopsy established metastasis, confirming stage IV disease with diffuse osteoblastic skeletal metastases. This case highlights tumor-mediated osteoblastic activity as an important endocrine mimic and underscores the need to integrate biochemical, radiologic, and histopathologic findings for the timely diagnosis of underlying malignancy.

## Introduction

Markedly elevated bone-specific alkaline phosphatase (BSAP) with radiographic skeletal abnormalities is commonly encountered in endocrine practice, often attributed to metabolic disorders with high bone turnover, including Paget disease of bone and osteomalacia. However, a similar biochemical phenotype can be generated by osteoblastic metastases through tumor-mediated osteogenesis. Bone metastases occurring in gastric carcinoma are typically osteolytic or mixed, with diffuse osteoblastic metastases being exceptionally rare [1]. When associated with diffuse osteosclerosis and biochemical features of high bone turnover, osteoblastic metastases can closely mimic metabolic bone disorders such as Paget disease of bone or osteomalacia, creating a diagnostic challenge. Herein, we report a case of poorly differentiated signet-ring cell gastric adenocarcinoma presenting with cardiac tamponade and synchronous osteoblastic skeletal metastases, initially evaluated as a metabolic bone disorder with high bone turnover.

## Case presentation

A 50-year-old woman presented with rapidly progressive dyspnea over 4 days, progressing to breathlessness at rest. Before the onset of these symptoms, she had a 2-month history of nonspecific gastrointestinal symptoms. The patient had no history of focal bone pain, skeletal deformity, chronic kidney disease, prior tuberculosis, known malignancy, family history of malignancy, or metabolic bone disease. On examination, oxygen saturation was 70% on room air, with pallor, pedal edema, elevated jugular venous pressure, and muffled heart sounds noted. Transthoracic echocardiography demonstrated a massive pericardial effusion with tamponade physiology. Following emergency pericardiocentesis with pigtail drainage, her symptoms improved.

## Diagnostic assessment

Pericardial fluid analysis revealed an exudative effusion, with pericardial fluid and serum protein levels of 6.51 and 6.6 g/dL, respectively, yielding a fluid-to-serum protein ratio of 0.98 (exudative: >0.5). Lactate dehydrogenase (LDH) in pericardial fluid was markedly elevated at 2880 U/L (SI [International System of Units]: 48.01 μkat/L) (reference range, <40 U/L [<0.67 µkat/L]) compared with serum LDH of 300 U/L (SI: 5 µkat/L) (reference range, 135-214 U/L [2.25-3.56 µkat/L]), with a fluid-to-serum LDH ratio of 9.6 (exudative: >0.6) and fluid LDH exceeding two-thirds of the upper limit of normal serum LDH. Furthermore, the fluid contained blood and had adenosine deaminase level of 25 U/L (SI: 0.42 µkat/L) (reference range, <40 U/L [<0.67 µkat/L]). Cytological examination revealed no malignant cells.

**Figure 1:**
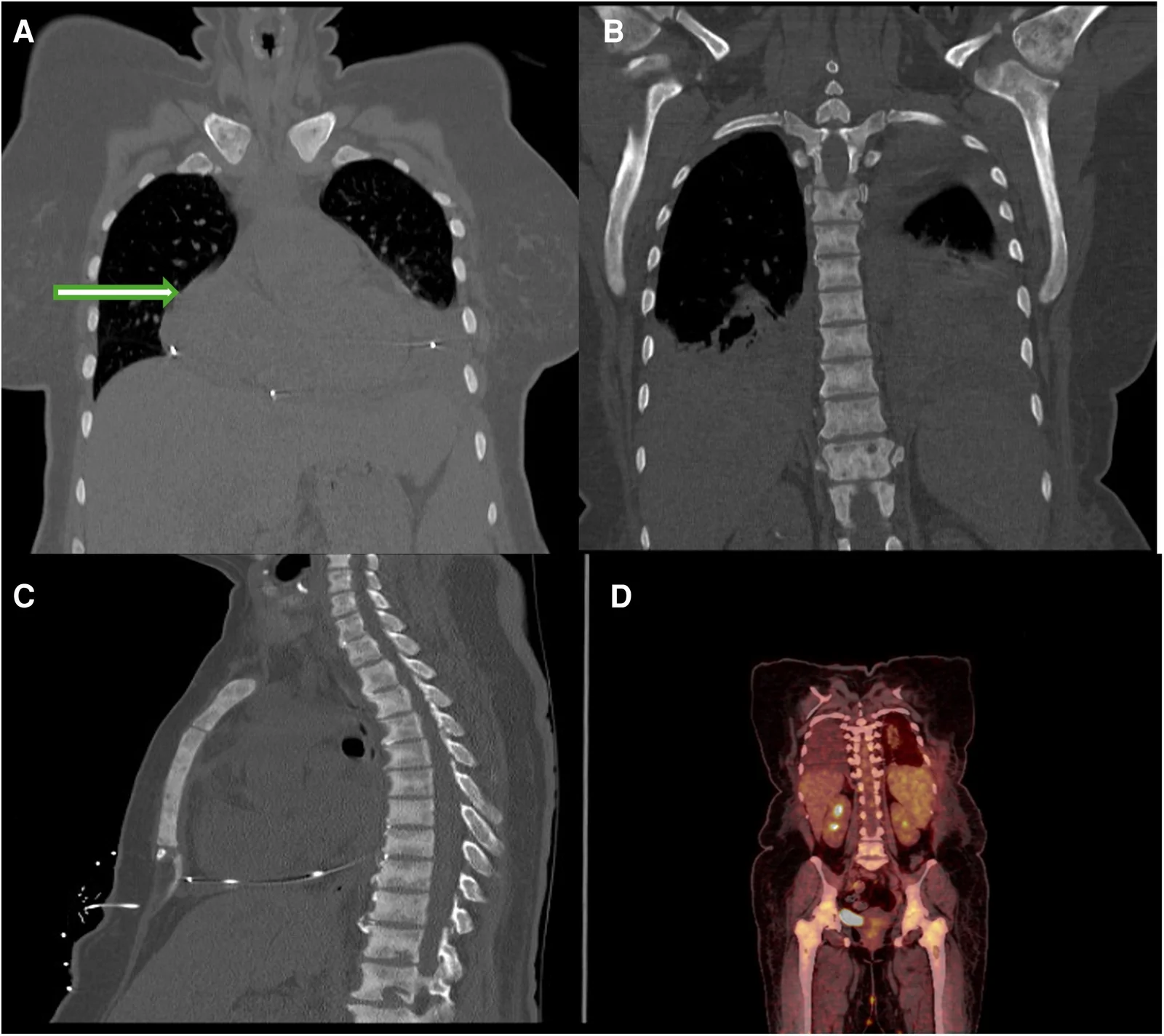
Osteoblastic skeletal involvement shown on computed tomography (CT) and fluorine-18 fluorodeoxyglucose positron emission tomography/computed tomography (^18^F-FDG PET/CT) (A) Coronal CT of the thorax reveals large pericardial effusion (arrow) reflecting cardiac tamponade physiology. (B) Coronal CT image of the thoracic spine shows diffuse and focal sclerotic lesions involving the vertebral bodies. (C) Sagittal reconstruction demonstrates uniform increase in vertebral density without cortical expansion or deformity. The imaging pattern of axial sclerosis without focal bone enlargement or lytic defects is atypical for primary metabolic bone disease, raising suspicion for osteoblastic metastatic involvement. (D) ^18^F-FDG PET/CT coronal fusion image reveals sclerotic vertebral lesions with mild metabolic activity but without a clearly hypermetabolic dominant primary lesion. The pattern suggests widespread skeletal infiltration; however, the primary gastric lesion showed limited FDG avidity, consistent with reduced metabolic activity observed in some diffuse-type gastric adenocarcinomas.

Chest computed tomography (CT) performed during the evaluation incidentally revealed multiple sclerotic lesions involving the vertebrae and ribs (Fig. 1A–C). The patient's markedly elevated BSAP (1437 IU/L (23.95 µkat/L; reference range, 12-50 IU/L [0.20-0.84 µkat/L])), combined with normal liver function tests, mild hypocalcemia (8.1 mg/dL (2.03 mmol/L); reference range, 8.6-10 mg/dL [2.1-2.6 mmol/L]), and secondary hyperparathyroidism (72 pg/mL (7.63 pmol/L); reference range, 15-65 pg/mL [1.6-6.9 pmol/L]), initially suggested a metabolic disorder with high bone turnover (Table 1). However, the radiologic pattern of multiple diffuse and focal axial sclerosis without deformity, cortical thickening, or bone expansion was atypical for Paget disease of bone. Given the absence of osteopenia or Looser zones on imaging, the relatively short duration of symptoms, and the predominant involvement of the axial skeleton, osteomalacia was considered unlikely. Moreover, mild hypocalcemia with elevated parathyroid hormone (PTH) indicated secondary hyperparathyroidism in the setting of severe vitamin D deficiency (25-hydroxyvitamin D, 5.9 ng/mL (14.7 nmol/L); reference range, >30 ng/mL [75-250 nmol/L]), although increased skeletal calcium uptake associated with high bone turnover from the underlying bone pathology may also have contributed (Table 1).

**Table 1:** Laboratory findings at presentation

Laboratory parameter	Reference range	Results
Hemoglobin	13-17 g/dL (8.1-10.6 mmol/L)	7.6 g/dL (4.72 mmol/L)
Total bilirubin	0.3-1.2 mg/dL (3-21 µmol/L)	1.11 mg/dL (18.8 µmol/L)
SGOT/SGPT	<32/<32 IU/L (<0.53/<0.53 µkat/L)	26/11 IU/L (0.433/0.182 µkat/L)
ALP	35-104 IU/L (0.58-1.73 µkat/L)	1592 IU/L (26.54 µkat/L)
Bone-specific ALP	12-50 IU/L (0.2-0.84 µkat/L)	1437 IU/L (23.95 µkat/L)
PTH	15-65 pg/mL (1.6-6.9 pmol/L)	72 pg/mL (7.63 pmol/L)
Calcium	8.6-10 mg/dL (2.15-2.50 mmol/L)	8.1 mg/dL (2.03 mmol/L)
Albumin	3.9-4.94 g/dL (39-50 g/L)	3.8 g/dL (38 g/L)
Corrected calcium	8.6-10 mg/dL (2.15-2.5 mmol/L)	8.26 mg/dL (2.07 mmol/L)
Phosphorus	2.5-4.5 mg/dL (0.81-1.45 mmol/L)	3.5 mg/dL (1.10 mmol/L)
Vitamin D	>30 ng/mL (75 nmol/L)	5.9 ng/mL (14.7 nmol/L)
LDH	135-214 U/L (2.25-3.57 µkat/L)	300 U/L (5 µkat/L)
FT4	0.93-1.7 ng/dL (10-23 pmol/L)	0.93 ng/dL (12 pmol/L)
TSH	0.27-4.2 µIU/mL (0.27-4.2 mIU/L)	1.630 µIU/mL (1.630 mIU/L)
Creatinine, serum	0.5-0.9 mg/dL (45-90 µmol/L)	0.9 mg/dL (80 µmol/L)
Sodium, serum	136-145 mEq/L (136-145 mmol/L)	133 mEq/L (133 mmol/L)
Potassium, serum	3.5-5.1 mEq/L (3.5-5.1 mmol/L)	3.5 mEq/L (3.5 mmol/L)
Chloride, serum	98-107 mEq/L (98-107 mmol/L)	99 mEq/L (99 mmol/L)
Bicarbonate, serum	22-29 mEq/L (22-29 mmol/L)	20 mEq/L (20 mmol/L)

Abbreviations: ALP, alkaline phosphatase; FT4, free thyroxine; LDH, lactate dehydrogenase; PTH, parathyroid hormone; SGOT, serum glutamic oxaloacetic transaminase; SGPT, serum glutamic pyruvic transaminase; TSH, thyroid-stimulating hormone.

Mild persistent anemia was also noted. Fluorine-18 fluorodeoxyglucose positron emission tomography/CT revealed multiple sclerotic skeletal lesions with mild, homogeneous marrow uptake; however, the radiologic pattern was nonspecific, and the primary source remained unidentified (Fig. 1D). Given the clinical complexity and inconclusive imaging, diagnostic testing and biopsy were undertaken to characterize the underlying disease mechanism.

**Figure 2:**
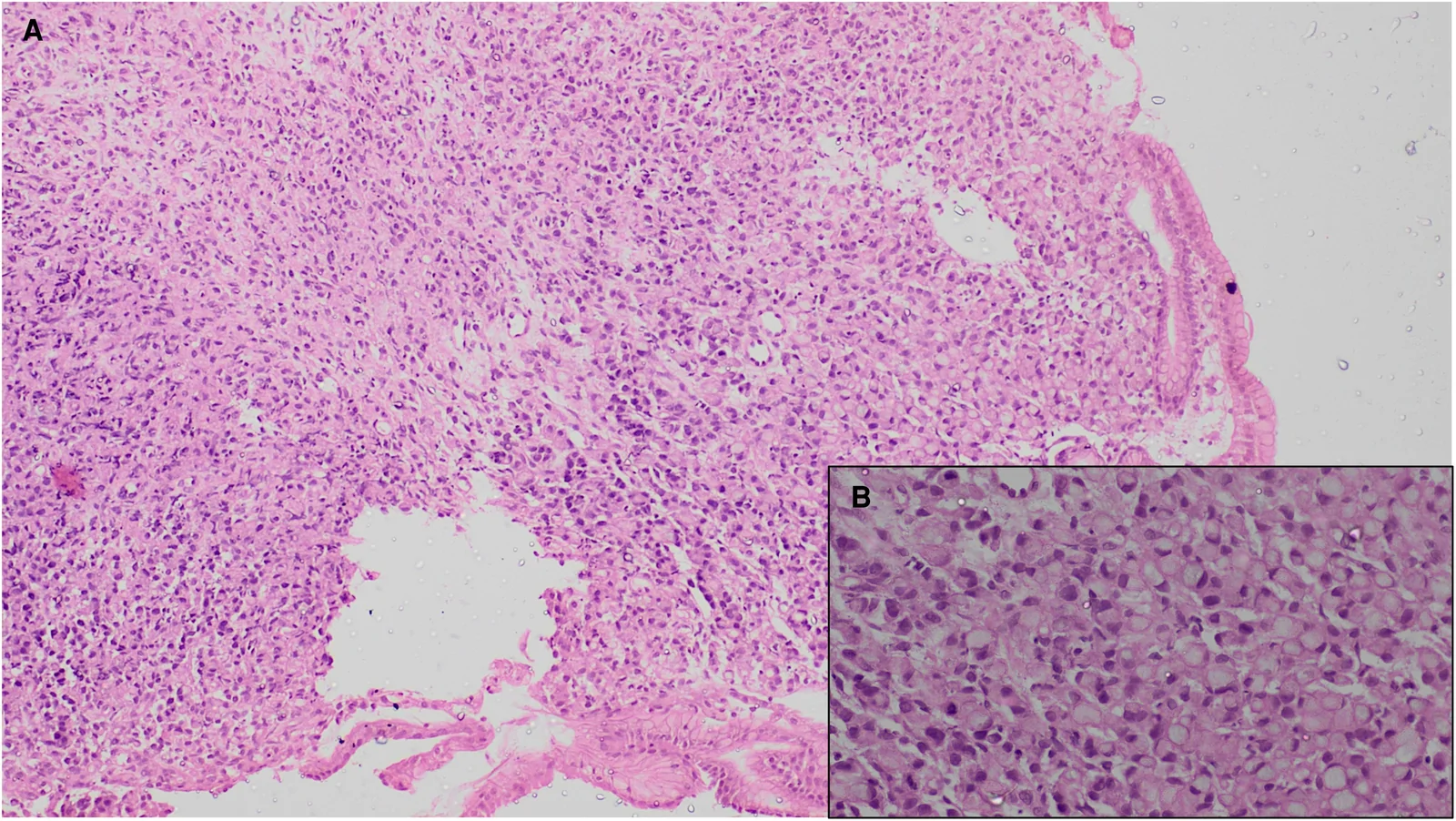
Hematoxylin–eosin staining of gastric nodule biopsy. (A) Original magnification (×200): gastric mucosa with submucosa containing atypical cells that resemble signet-ring cells. (B) Higher magnification (×400): Sheets of malignant cells with abundant intracellular mucin that displaces nuclei peripherally, consistent with signet-ring cell morphology (arrow). These findings confirm poorly differentiated signet-ring cell adenocarcinoma.

**Figure 3:**
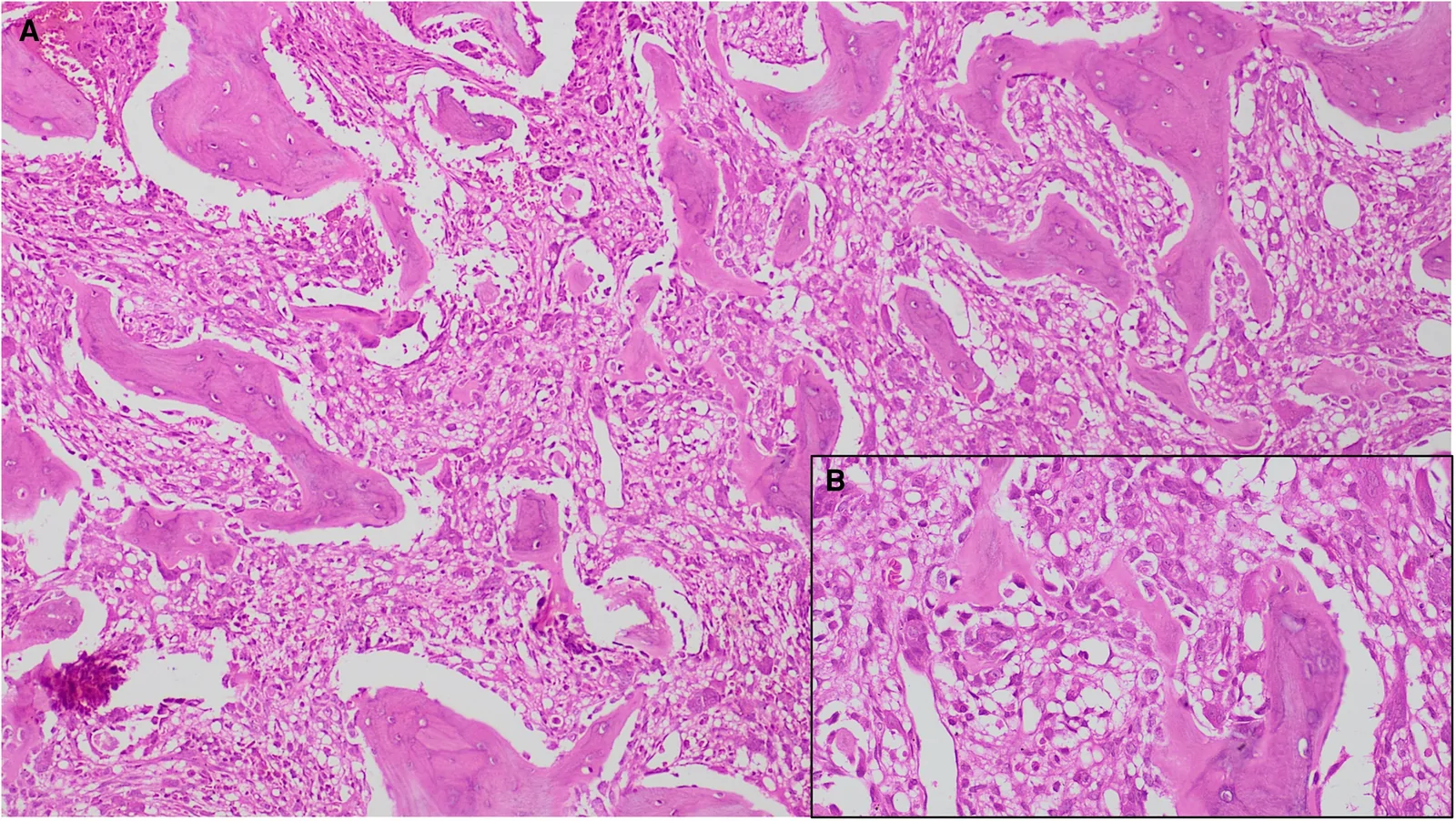
Bone marrow biopsy. (A) Hematoxylin–eosin staining (original magnification ×200) showing bony trabecula enclosing marrow spaces with atypical cells. (B) Higher magnification (×400) highlights signet-ring cells with intracellular mucin and displaced nuclei. The morphologic features align with metastatic signet-ring cell adenocarcinoma involving bone marrow, confirming stage IV disease with diffuse osteoblastic skeletal metastases.

Considering the persistent gastrointestinal symptoms, the patient underwent upper gastrointestinal endoscopy, which showed an irregular lesion in the gastric antrum. Histopathology confirmed poorly differentiated signet-ring cell adenocarcinoma (Fig. 2). The skeletal abnormalities were directly evaluated by bone marrow biopsy of a sclerotic vertebral site, which revealed nests of atypical epithelial cells with signet-ring morphology and positive pancytokeratin expression, concordant with gastric origin (Fig. 3). These findings established metastatic infiltration, confirming stage IV cancer (T4N0M1).

## Treatment

Considering the diffuse osseous involvement and heightened bone turnover, palliative chemotherapy and antiresorptive therapy were recommended. Severe vitamin D deficiency was treated to mitigate hypocalcemia and reduce the risk of metabolic complications, particularly in the context of potential antiresorptive therapy.

## Outcome and follow-up

The patient had declined all cancer-directed and bone-targeted therapies, accepted only vitamin D supplementation, and died within 3 weeks of diagnosis.

## Discussion

In adults, osteosclerotic skeletal lesions with elevated bone turnover markers warrant evaluation for metabolic bone disease, particularly Paget disease of bone and osteomalacia. At the same time, nonendocrine causes, including marrow infiltrative disorders and malignancy, should also be considered. Although initially considered because of markedly elevated BSAP levels, Paget disease of bone was deemed unlikely because of the absence of asymmetric skeletal changes, deformity, or bone pain [2]. Myeloproliferative disorders may yield marrow abnormalities but are not associated with marked BSAP elevation, as observed in the present case [3]. Osteomalacia was improbable, given the existence of diffuse sclerosis rather than osteopenia or pseudofractures, as well as the short symptom duration [4]. Conversely, osteoblastic metastases demonstrate tumor-mediated osteoblast recruitment and aberrant activation of osteogenesis, resulting in increased osteoid deposition and mineralization. However, these features were not evident in our biopsy specimen, which showed osteoclast-like giant cells rather than overt osteoblastic proliferation. Such absence may be explained by sampling from a single skeletal site and the inherent heterogeneity of metastatic bone involvement. Therefore, the osteoblastic nature was inferred from diffuse osteosclerosis on imaging and markedly elevated BSAP. While classically associated with prostate carcinoma, osteoblastic metastases are rarely described in poorly differentiated gastrointestinal malignancies, including diffuse-type gastric adenocarcinoma [5].

The patient's biochemical profile—markedly elevated BSAP, increased LDH, anemia, and relative hypocalcemia—reflects extensive skeletal remodeling and marrow replacement. Hypocalcemia in osteoblastic metastases likely reflects increased skeletal sequestration of calcium during osteoblastic bone formation, similar to hungry bone syndrome's rapid skeletal calcium uptake [6]. This pattern suggests that tumor-mediated osteogenic signaling has predominated over osteoclastic activity, resulting in a net sclerotic phenotype. Experimental studies suggest that activation of Wnt/β-catenin signaling and tumor–stromal interactions may promote osteoblast activation, although mechanistic confirmation was unavailable [7]. Measuring 1,25-dihydroxyvitamin D (or calcitriol) could have provided further mechanistic insights, considering that elevated PTH would stimulate renal 1-alpha hydroxylase activity and increase calcitriol production to compensate for hypocalcemia and vitamin D deficiency. However, this investigation could not be conducted because of financial constraints. Persistent hypocalcemia despite this expected compensatory response would further support ongoing calcium sequestration within osteoblastic lesions [8].

Diffuse-type (signet-ring cell) gastric adenocarcinoma exhibits infiltrative growth, early lymphovascular invasion, and a propensity for peritoneal and hematogenous dissemination [9]. In some diffuse-type tumors, reduced fluorodeoxyglucose avidity has been attributed to lower glucose transporter 1 expression [10]. This finding might explain the limited metabolic conspicuity of the primary lesion on positron emission tomography in the present case despite widespread skeletal involvement. Additionally, the patient had marrow infiltration, which confirmed stage IV cancer and accounted for her anemia and rapid clinical decline. Concurrently, the presence of malignant cardiac tamponade and diffuse osteoblastic skeletal metastases suggests widespread metastases and aggressive tumor biology [11].

Malignant pericardial effusion commonly occurs when the tumor involves the pericardium, often seen in advanced disease and showing a poor prognosis. Direct invasion, lymphatic dissemination, or hematogenous spread allows tumor cells to reach the pericardium, causing vascular disruption and fluid or blood accumulation within the pericardial space. Less commonly, tumor involvement of mediastinal lymph nodes can obstruct cardiac lymphatic drainage, leading to pericardial effusion, which typically presents as transudative and may have negative cytology [12]. In our patient, while cytology was negative for malignant cells, pericardial fluid analysis revealed a hemorrhagic exudative effusion. Nonetheless, malignancy-related pericardial effusion could not be ruled out because cytology has variable sensitivity (57-100%), which may cause false-negative results [13]. The presence of disseminated metastatic disease with massive pericardial effusion causing tamponade in our patient strongly supported malignancy-associated tamponade physiology. While pericardial biopsy may improve diagnostic sensitivity, pericardial effusions in advanced malignancy have been linked to poor prognosis; therefore, rather than conducting further invasive diagnostic evaluation, urgent relief from tamponade and symptom control were prioritized.

The number of reported cases of osteoblastic skeletal metastases from gastrointestinal cancers remains limited, although such cases are increasingly recognized with improved imaging and biochemical profiling. Although bone metastases from gastric cancer are uncommon in routine clinical practice (reported clinical frequencies: 0.9-20%), rates reported in autopsy series and dedicated bone metastasis screening studies are higher [14, 15]. Our case demonstrates that diffuse-type gastric adenocarcinoma may present with an osteosclerotic phenotype and markedly elevated BSAP, closely resembling Paget disease of bone or osteomalacia. This underscores the need to consider metastatic disease in patients with unexplained osteosclerosis, and disproportionate elevation of bone turnover markers. Reduced fluorodeoxyglucose avidity may further underestimate disease burden, emphasizing the importance of prompt tissue diagnosis. The coexistence of diffuse osteoblastic metastases and malignant cardiac tamponade highlights the disease's aggressive nature and warrants a high index of suspicion in atypical presentations.

## Learning points

- Multiple osteosclerotic bone lesions with elevated bone turnover markers should prompt evaluation for metastatic disease even without a known primary tumor.
- Gastric adenocarcinoma, particularly diffuse-type histology, should be considered as a differential diagnosis for osteoblastic metastases.
- Solely relying on metabolic imaging may underestimate disease burden in tumors with low fluorodeoxyglucose avidity; therefore, tissue diagnosis is important in nonspecific imaging findings.
- Early recognition of atypical metastatic patterns is essential for accurate staging, appropriate counseling, and timely initiation of systemic therapy and bone-targeted treatment to mitigate skeletal-related complications [16].

## Contributors

All authors made individual contributions to authorship. S.M.M., A.R., and S.S. were involved in the diagnosis and management of the patient. L.D.J. was involved in the histopathology section and preparation of histology images. V.S.P.M. was involved in the radiological diagnosis. A.R. and V.D. contributed to the analysis and interpretation of the clinical data and critically revised the manuscript for important intellectual content. All authors reviewed and approved the final draft.

## Data Availability

Data sharing is not applicable to this article as no datasets were generated or analyzed during the current study.
